# Community Analysis of Arbuscular Mycorrhizal Fungi in Roots of *Poncirus trifoliata* and *Citrus reticulata* Based on SSU rDNA

**DOI:** 10.1155/2014/562797

**Published:** 2014-08-05

**Authors:** Peng Wang, Yin Wang

**Affiliations:** ^1^Institute of Citrus Research, Zhejiang Academy of Agricultural Sciences, Taizhou 318026, China; ^2^National Center for Citrus Variety Improvement, Zhejiang Branches, Taizhou 318026, China

## Abstract

Morphological observation of arbuscular mycorrhizal fungi (AMF) species in rhizospheric soil could not accurately reflect the actual AMF colonizing status in roots, while molecular identification of indigenous AMF colonizing citrus rootstocks at present was rare in China. In our study, community of AMF colonizing trifoliate orange (*Poncirus trifoliata *L. Raf.) and red tangerine (*Citrus reticulata* Blanco) were analyzed based on small subunit of ribosomal DNA genes. Morphological observation showed that arbuscular mycorrhizal (AM) colonization, spore density, and hyphal length did not differ significantly between two rootstocks. Phylogenetic analysis showed that 173 screened AMF sequences clustered in at least 10 discrete groups (GLO1~GLO10), all belonging to the genus of* Glomus* Sensu Lato. Among them, GLO1 clade (clustering with uncultured Glomus) accounting for 54.43% clones was the most common in trifoliate orange roots, while GLO6 clade (clustering with *Glomus intraradices*) accounting for 35.00% clones was the most common in red tangerine roots. Although, Shannon-Wiener indices exhibited no notable differences between both rootstocks, relative proportions of observed clades analysis revealed that composition of AMF communities colonizing two rootstocks varied severely. The results indicated that native AMF species in citrus rhizosphere had diverse colonization potential between two different rootstocks in the present orchards.

## 1. Introduction

The majority of terrestrial plants have symbioses with arbuscular mycorrhizal fungi (AMF) which can benefit their host plants in several ways, enhancing mineral nutrients uptake, especially phosphate, improving water status, protecting from pathogens [1, 2], and facilitating rhizospheric soil structure formation and maintenance included [3, 4]. Thereby, AMF are perceived as one of the most important components of various ecosystems. It is well known that AMF have a broad host range, but some studies have revealed that each individual fungus is functionally distinct [5, 6]. Furthermore, AMF community with different species composition could induce different growth response in plants and play a potential role to determine ecosystem variability and productivity [7]. Thus, the native AMF species colonizing a given host plant in field should be clear at the community level.

The traditional method of AMF identification relies extensively on the morphological and developmental characteristics of fungal spore or hyphae; however, morphological methods have some flaws. The species of none or few spore-bearing AMF are often neglected in field soil investigation, and spore morphotyping requires considerable experience [6, 8]. In addition, the common AMF species in soil have various degrees host's selectivity. Thus, this method cannot reflect the actual status of AMF species colonizing plant roots [9]. In last few decades, the molecular identification approaches based on the small subunit of ribosomal DNA (SSU rDNA) analysis offered considerable utility to identify distantly related species or groups of related AMF [10]. Recently, the method of amplifying a portion SSU rDNA of AMF targeted by improved primers AML1 and AML2 from whole root or spore DNA, then followed by cloning and sequencing, was used for AMF community research, and the new primers with much better specificity could amply all published AMF sequences except those from* Archaeospora trappei* [11].

Citrus is one of the most economically fruit crops in China indeed in the world. Trifoliate orange (*Poncirus trifoliata *L. Raf.) and red tangerine (*Citrus reticulata* Blanco), two widespread citrus rootstocks in hilly orchards, southern China, depend on AMF greatly to improve nutrition absorption and water status [12–14]. AMF have been proposed as a potent biofertilizer in organic agriculture in China. However, the description of the community of AMF in citrus field systems was rare in the last few years, particularly the molecular identification of indigenous AMF colonizing citrus rootstocks at present in China. Hence, in this field survey, the community of AMF colonizing trifoliate orange and red tangerine was studied by analyzing the SSU rDNA gene of AMF in hilly sod culture orchards.

## 2. Materials and Methods

### 2.1. Site Description and Sampling

Our investigation was carried out in citrus specimen orchards located at hill slopes in Wuhan (29°58′–31°22′N, 113°41′–115°05′E), Southern China. This area has a semitropical monsoon climate with annual sunlight of 1810–2100 h, frost-free period of about 211–272 days, mean precipitation of 1269 mm, and mean temperature of 15.8–17.5°C. In citrus orchards, the soil management practice of planting 2 rows of bahia grass (*Paspalum notatum* Flügge) between trees, mowing grass to control grass height, and mulching grass under trees has been applied continuously for 5 years. The biological organic fertilizers (7% N, 4% P_2_O_5_, 4% K_2_O, and 20% organic matter) were used in all orchards to preserve the basic soil fertility. The orchard soil was classified as yellow sandy clay soil (Acrisols in FAO Taxonomy).

Six plots where citrus trees (Satsuma Mandarin), respectively, were grafted on trifoliate orange (3 replicated plots, T1~T3) or red tangerine (3 replicated plots, R1~R3) were selected in citrus orchards. Five healthy citrus trees were randomly sampled in each plot. We simultaneously collected fine roots (Φ ≤ 1 mm) and rhizospheric soils (about 1 kg) of one single tree from four directions (east, west, south and north) at a depth of 0–30 cm after removing upper covering within the dripping line of the tree canopy in April 2009. The roots were taken to the laboratory, carefully washed with tap water to remove soil, and then chopped into 1 cm long segments. One subsamples were fixed in FAA (formalin/acetic acid/ethanol, 13/5/200, v/v/v) solution for 24 h then stored at 4°C, and the other subsamples were immediately frozen using liquid nitrogen and stored at −80°C until molecular analysis. To evaluate AM fungal spores and hyphae, soil was air-dried for 2 weeks, carefully ground by hands, passed through a 2 mm mesh screen, and stored at 4°C until analysis.

### 2.2. Assessment of Main Soil Chemical Properties

Soil organic matter (OM) was measured by the procedure of K_2_CrO_7_-H_2_SO_4_ humid oxidation, alkali-hydrolysable N (AN) by the alkaline hydrolysis diffusion method [15]. Available P (AP) was extracted with NaHCO_3_ following the Olsen method [16] and determined with spectrophotometer (UV-2450, Shimadzu, Japan) by reacting with (NH_4_)_2_MoO_4_ using ascorbic acid as a reductant in the presence of antimony method of Murphy and Riley [17]. Available K (AK) was extracted with NH_4_HCO_3_ + DTPA (diethylenetriaminepentaacetic acid) and analyzed using an ICP-AE spectrometer. Soil pH was determined using a suspension of the soil sample in water at a ratio of 1 : 2.5 (w/v) with a Mettler Toledo 320 pH meter (Mettler-Toledo Ltd., Switzerland).

### 2.3. Assessment of AM Colonization, Spore Density, and Hyphal Length

Colonization of various AM fungal structures in citrus roots was examined according to Koske and Gemma [18] under a compound-light microscope (Olympus-BH-2, Tokyo, Japan). AM fungal colonization rate was estimated using the magnified intersection method [19]. The ratio of root length with total AM colonization (RLT), arbuscules (RLA), and vesicles (RLV) were quantified by examining 200 intersections per sample. Spores were extracted from soils using the wet sieving and sucrose gradient centrifugation technique with minor modification [20], and the total number was counted with the stereoscopic microscope (Tech-XTS-30, Beijing, China). Spore density (SD) was expressed as the number of spores and sporocarps per 100 g dry soil. Soil hyphal length (HL) was determined according to Bethlenfalvay and Ames [21], with the aid of an ocular micrometer under a compound-light microscope, and the lengths of hyphae per 1 g dry weight of soil were calculated.

### 2.4. DNA Extraction and Nested PCR

Total genomic DNA was extracted from roots according to the method proposed by Saito et al. [22]. The quality and quantity of DNA from root samples were checked on a 1.0% agarose gel and then stored at −20°C, serving as templates for the polymerase chain reaction (PCR). Partial SSU rDNA gene fragments were amplified using nested PCR with the universal eukaryotic primers NS1 and NS4 [23] on a Bio-Rad PCR Thermal Cycler, Model S1000 (Bio-Rad, California, USA). The first PCR product was diluted 100 times with 1 × TE (1 M Tris-HCl, 0.5 M EDTA, pH 8.0) buffer and 2.5 *μ*L was used as template DNA in second round PCR reaction performed using the specific primers AML1 and AML2 [11].

### 2.5. Cloning and Sequencing

The second PCR products were cut out and DNA was extracted with a gel DNA Purification Kit according to the manufacturer's instructions (Takara, Dalian, China), cloned into pMD18-T Simple Vector (Takara, Dalian, China) following the manufacturer's protocol, and transformed into competent Escherichia coli DH-5*α*. Plasmid clones were identified based on blue-white screening. Approximately 30 transformants were randomly selected for each sample of two rootstocks and stored in 20% glycerol at −20°C and bidirectional sequencing was performed by using vector primers M13 at Sangon Biological Engineering Technology and Service Co., Ltd. (Shanghai, China).

### 2.6. Reconstruction of Phylogenetic Tree

The resulting sequences were edited using the BioEdit program (Version 7.0.9) and the obtained sequences similarities were determined using the BLAST sequence similarity search tool provided by GenBank. Before processing the sequences for phylogenetic analysis, representative sequences were targeted to define the divergent sequences from the same species by using the DOTUR program [24]. The distance matrix was determined by the DNAdist program in the PHYLIP package (Version 3.69), and then the rarefaction curves were created by using the DOTUR program. According to the results, the representative sequences and reference sequences obtained from GenBank were aligned by using the Clustal X (Version 1.83), and* Mortierella polycephala*, the species of Zygomycota, which is a sister group to Glomeromycota [25], was used as the out-group. The neighbour-joining analyses were performed for the aligned data sets by Clustal X with bootstrap analyses of 1,000 replications in PAUP (version 4.0b10). The neighbor-joining trees were displayed using TreeView.

### 2.7. Statistical Analysis

Shannon-Wiener index (*H*′) and relative proportion of each clade were used to characterize diversities between the communities of AMF colonizing two different rootstocks [26], using the formula: *H*′ = −Σ*p*_*i*_ln⁡*p*_*i*_, where *p*_*i*_ is the frequency of the *i*th clade. All data were subjected to analysis of variance using SAS statistical software (Version 9.1). Means were compared by least significant differences (LSD) at the 0.05 level.

### 2.8. Nucleotide Sequence Accession Numbers

The SSU gene sequences reported in this study have been deposited in GenBank under accession numbers JQ350740 to JQ350804. Only representative sequences were deposited.

## 3. Results

### 3.1. Soil Chemical Properties

In our study, the content of soil OM ranged from 12.20 to 12.76 g kg^−1^ dry soil, AN from 69.00 to 72.13 mg kg^−1^, AP from 13.40 to 14.70 mg kg^−1^, AK from 165.89 to 166.85 mg kg^−1^, and pH from 5.78 to 5.84 in trifoliate orange sampled plots; OM from 11.24 to 12.08 g kg^−1^ dry soil, AN from 65.34 to 68.65 mg kg^−1^, AP from 13.43 to 15.17 mg kg^−1^, AK from 152.52 to 155.96 mg kg^−1^, and pH from 5.78 to 5.82 in red tangerine sampled plots. The citrus rhizospheric soil chemical property tested results showed no significant differences among different sampled plots from experimental orchards (Table 1).

### 3.2. Arbuscular Mycorrhizal Colonization, Spore Density, and Hyphal Length

**Figure 1:**
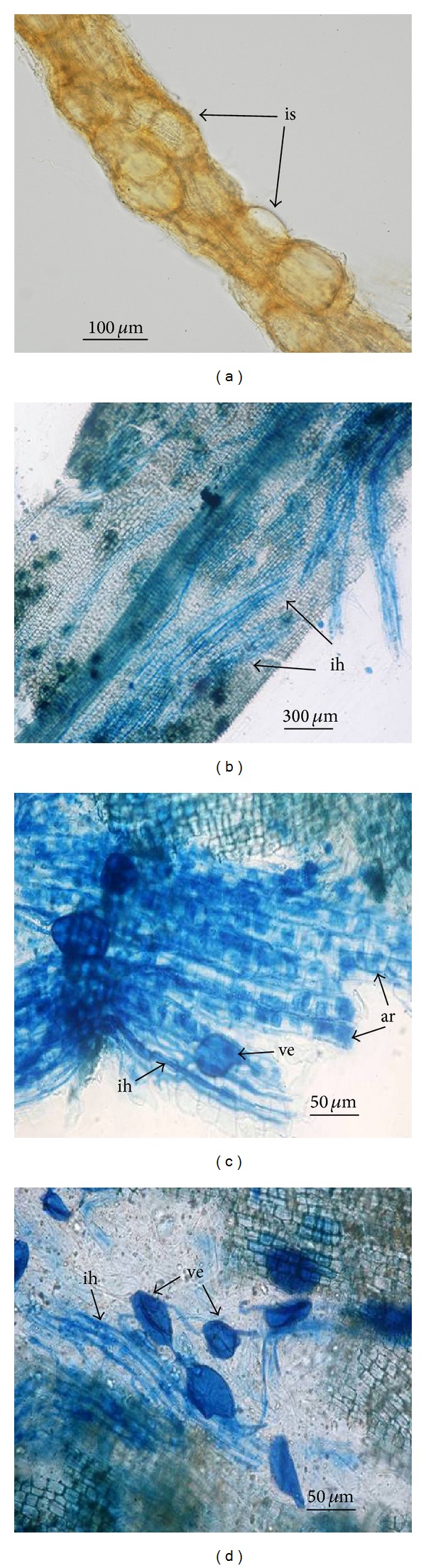
Intraradical spores (is), intercellular hyphae (ih), arbuscules (ar), and vesicles (ve) structures were observed in citrus roots. (a) Intraradical spores in citrus root, bar = 100 *μ*m; (b) AM colonization citrus root, bar = 300 *μ*m; (c) intercellular hyphae, arbuscules, and vesicles in citrus root, bar = 50 *μ*m; (d) intercellular hyphae and vesicles in citrus root, bar = 50 *μ*m.

In the present study, all citrus rootstocks surveyed were colonized by native AMF and formed typical AM structures including intra- and intercellular hyphae, arbuscules, and vesicles (Figure 1). Occasionally, intraradical spores were observed alone or together in the root tissues. The AMF hyphae in citrus roots were prevalent in all samples. The arbuscules were abundant and sometimes occurred in clusters; however, the vesicles were less observed in citrus roots.

**Figure 2:**
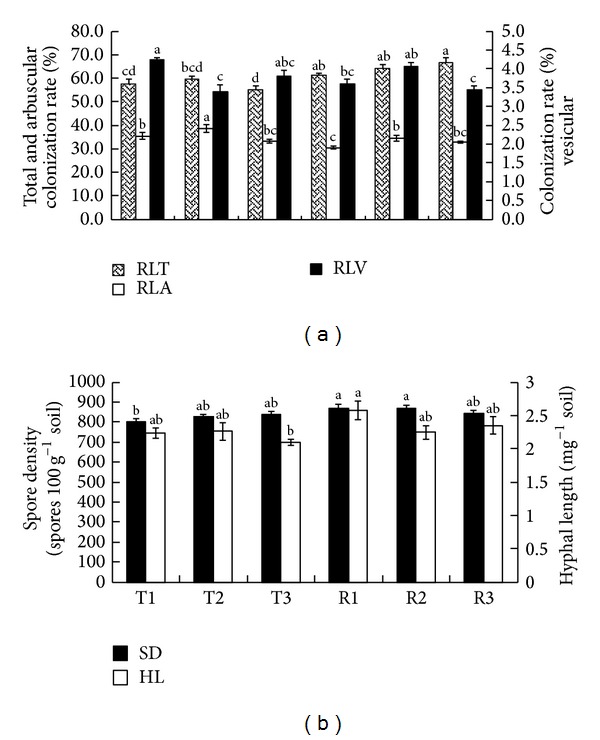
AM colonization rates in citrus roots, spore density, and hyphal length in trifoliate orange (T) and red tangerine (R) rootstocks orchards. (a) The ratio of root length with arbuscules (RLA), vesicles (RLV), and total AM colonization (RLT); (b) spore density (SD) and hyphal length (HL). Bars with the different letters show significant differences at the 0.05 level.

The RLT of trifoliate orange roots ranged from 55.10% to 59.58%, RLA from 33.09% to 38.54%, and RLV from 3.40% to 4.23%. In trifoliate orange rhizospheric soils, SD ranged from 802 to 838 spores 100 g^−1^ soil and HL from 2.09 to 2.26 m g^−1^ soil. The RLT of red tangerine roots ranged from 61.08% to 66.48%, RLA from 30.44% to 34.55%, and RLV from 3.43% to 4.07%. In red tangerine rhizospheric soils, SD ranged from 843 to 870 spores 100 g^−1^ soil and HL from 2.24 to 2.57 m g^−1^ soil (Figure 2). Overall, except RLT, the functionality of AMF species prevalent within the roots and rhizosphere of two divergent rootstocks did not differ severely.

### 3.3. Molecular Phylogenetic Analysis

**Figure 3:**
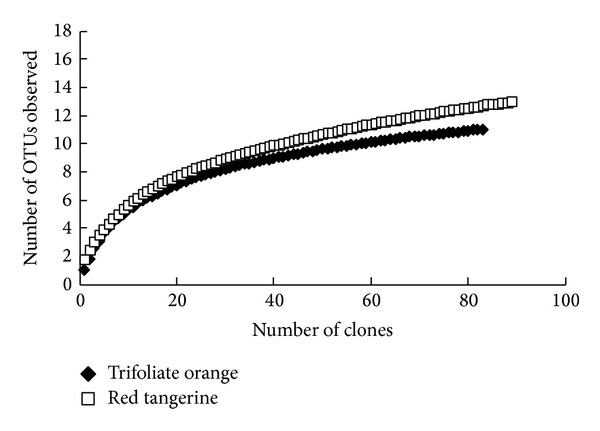
Rarefaction curves using AM fungal SSU rDNA sequences from roots of trifoliate orange and red tangerine in orchards.

In our study, partial SSU rDNA of AMF colonizing trifoliate orange and red tangerine was successfully amplified by the primer pairs AML1 and AML2. Among total of 180 randomly clones, 173 (96.1%) products of the expected size ranging from 792 to 799 bp in length were sequenced. Additionally, only 7 non-Glomeromycota PCR products, having 793 bp, from the root samples of trifoliate orange were cloned and excluded from further analysis. The distance matrix result showed that 11 and 13 OTUs (Operation taxonomic unit, less than 97% sequence similarity) were contained in SSU rDNA clone libraries of AMF colonizing two rootstocks, respectively. The generated rarefaction curves showed the curves became flatter when the number of the clones rose to 60 (Figure 3).

**Figure 4:**
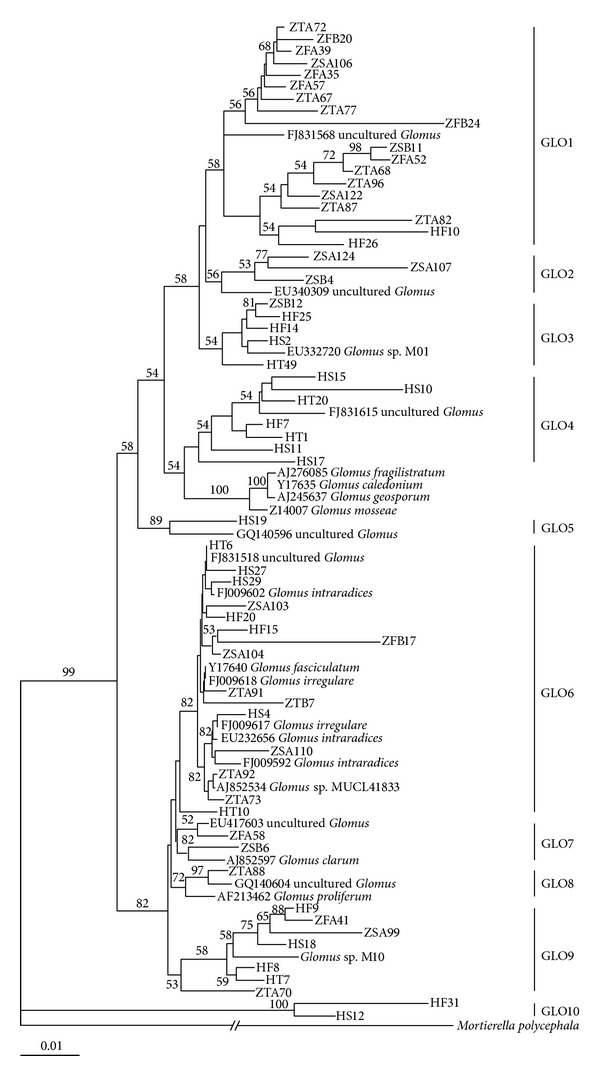
Neighbor-joining tree derived from the AML1-AML2 SSU rDNA sequences from AM fungi colonizing roots of trifoliate orange and red tangerine in orchards. The clone initial letter “Z” represents trifoliate orange and “H”: red tangerine, respectively. The rest of the letters and numbers were clone coded representation. Others were reference sequences searched in GenBank. Bootstrap values were calculated from 1000 replications.

**Figure 5:**
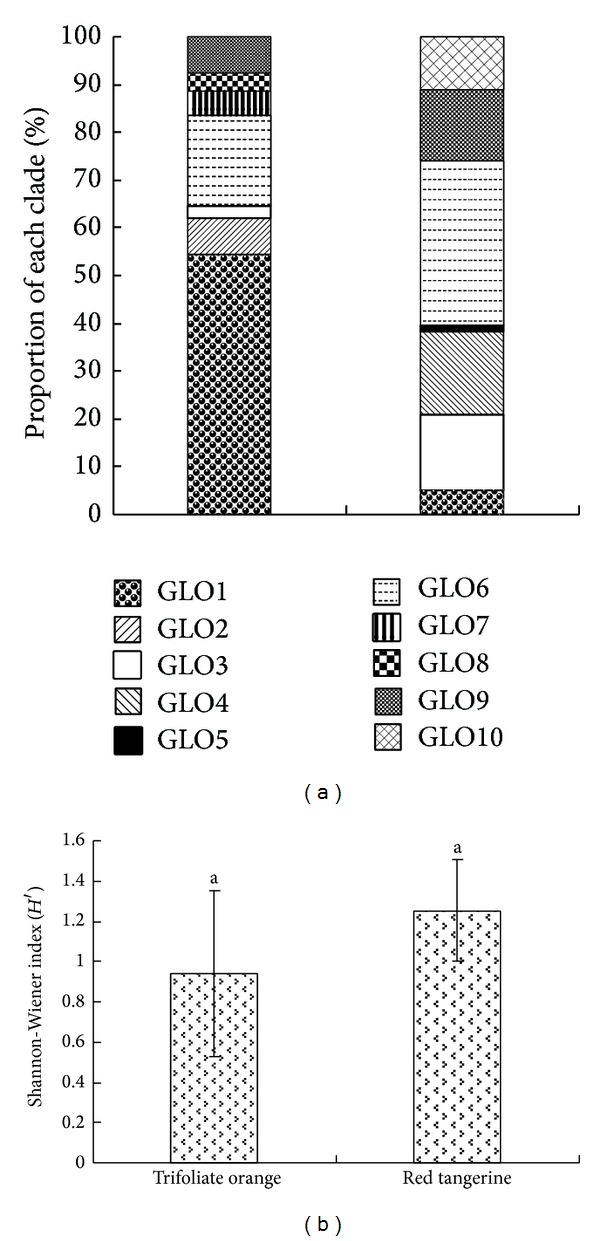
Bar plot showing the relative proportions (a) and Shannon-Wiener index (b) of the observed clades in the two AM fungal analyzed libraries of trifoliate orange and red tangerine. Bars with the same letter are not significantly different at the 0.05 level.

A dataset containing 61 representative sequences and 22 reference sequences obtained from GenBank representing the* Glomus* (*G*.) Sensu Lato species was constructed. The phylogenetic tree exhibited that the obtained AM fungal SSU rDNA sequences were separated into at least 10 discrete sequence groups (Figure 4). Of AMF community colonizing trifoliate orange roots, the majority of clones (54.43%) fell into the GLO1 clade; however, none of the clones could cluster with morphospecies in this clade. While, the most clones (35.00%) of AMF community colonizing red tangerine roots fell into the GLO6 clade clustering with* G. intraradices*,* G. fasciculatum,* and* G. irregulare*. The AMF clones falling into GLO2, GLO7, and GLO8 clades only colonized trifoliate orange. Among them, GLO7 clustered with* G*.* clarum* and GLO8 with* G*.* proliferum*. The AMF clones falling into GLO4, GLO5, and GLO10 clades only colonized red tangerine. The AMF clones falling into the GLO1, GLO3, GLO6, and GLO9 clades did not only colonized trifoliate orange but also red tangerine. By statistics analysis, we could further find that the relative proportions of AMF clades colonizing both trifoliate orange and red tangerine varied greatly (Figure 5(a)). Nevertheless, the Shannon-Wiener index of the AMF communities colonizing two rootstocks showed no significant difference (Figure 5(b)).

## 4. Discussion

Arbuscular mycorrhizal symbiosis between modern plants and fungi is ubiquitous [2]. Wu et al. [27] reviewed that a total of 45 AMF species belonging to seven genera such as* Acaulospora*,* Entrophospora*,* Gigaspora*,* Glomus*,* Pacispora*,* Sclerocystis*, and* Scutellospora* were found in citrus rhizosphere. Species of genera such as* Acaulospora*,* Gigaspora*, and* Glomus* were dominantly observed in citrus rhizosphere. In our previous study, 18 AMF morphological species belonging to 5 families, for example,* Acaulosporaceae* (4 species),* Claroideoglomeraceae* (2 species),* Gigasporaceae* (1 species),* Glomeraceae* (9 species), and* Pacisporaceae *(2 species) were observed in trifoliate orange rhizospheric soils, and 18 AMF species belonging to 6 different families, for example,* Acaulosporaceae* (4 species),* Archaeosporaceae* (1 species),* Claroideoglomeraceae* (2 species),* Gigasporaceae* (2 species),* Glomeraceae* (8 species), and* Pacisporaceae* (1 species) in red tangerine rhizospheric soils [28, 29]. However, the present molecular data showed that native AMF colonizing two citrus rootstocks all belonged to the genus of* Glomus* Sensu Lato separated into 10 clades in our study. Some studies also reported that AMF community identified by molecular approaches in roots all belonged to* Glomus* different from morphologically identified species community in rhizospheric soils [9, 30]. This may imply that some AMF species in citrus rhizosphere do not colonize citrus roots but grass roots in field orchards further indicate that AMF species in soil have various degrees host's selectivity. At morphological observation level, we found that the integrated mycorrhizal status with respect to colonization rate in citrus roots, spore density, and hyphal length in citrus rhizosphere showed no notable differences between the two rootstocks. This might be attributed to long-term application of same agricultural practices like sowing bahia grass in the all surveyed orchards.

AMF in the surveyed orchards exhibited diverse colonization potential between trifoliate orange and red tangerine in our study. It is well known that trifoliate orange tolerance is clearly different from red tangerine in response to water, nutrition, temperature stresses, and so on. Thus, this result was in accord with that AMF species colonization exhibited changes depending on functional interactions with their hosts [31, 32]. Composition of AMF changed heavily between habitat types and host species, which could result in notable differences in AMF alleviating various stresses [33, 34]. So, it was necessary to research the AMF community in order to identify the dominant species colonizing the given citrus rootstock under specific environment conditions. However, the AMF species diversity index indicated no significant differences between the two rootstocks in our study. This might be due to sowing the mycorrhizal plant of bahia grass as the sod between citrus trees in all experimental orchards. Some studies reported that increasing plant species richness was correlated with changes in AMF community composition, and mycorrhizal weeds like bahia grass maintained in orchards were favourable for AMF propagation and mycorrhizal symbiosis formation with citrus trees [35, 36].

In the present study, partial sequences were related to the* G*.* intraradices*,* G*.* fasciculatum,* and* G*.* clarum* which have a global distribution and comprise the common species of AMF in various ecosystems [37]. Indeed, numerous scientific experimental studies have documented that most agricultural crops benefited from their inoculations [38]. Our molecular data also showed that red tangerine was most colonized by these AMF species, and Nemec et al. [39] reported that* G*.* fasciculatum* was consistently associated with young citrus trees (0~30 yr). This suggested that it was suitable to choose the* G. intraradices*,* G. fasciculatum*, or* G. clarum *as broad spectrum soil inoculants in citrus nursery. However, it should be mentioned that the corresponding morphospecies with the GLO1 clade is more desirable to be identified and isolated as AM inoculants in the future study due to their quite ability to establish symbiosis with the trifoliate orange in this study.

In the present study, the most of AM fungal sequences without corresponding morphospecies recorded in the International Nucleotide Sequence Database Collaboration (INSDC) indicated that the actual AMF diversity reflected by the traditional morphospecies was underestimated [40], and a considerable number of taxa are rarely reported in citrus orchards. Interestingly, it was found that no related AM fungal sequence type to GLO10 deposited in INSDC. The result indicated that GLO10 might be a type of novel *G* spp. or specific to these experimental citrus orchards.

## 5. Conclusions

Native AMF colonizing roots of trifoliate orange and red tangerine was first analyzed based on SSU rDNA gene in field orchards in China. Phylogenetic results showed that AMF colonizing two rootstocks all belonged to the genus of* Glomus* Sensu Lato, and some native AMF could establish symbiosis with both rootstocks in the sod culture orchards. However, molecular evidence also revealed that native AMF showed various degrees host's selectivity between trifoliate orange and red tangerine in citrus orchards.

## Figures and Tables

**Table 1:** Chemical properties of the experimental soils from trifoliate orange (T) and red tangerine (R) sampling plots in citrus orchards.

Sites	Soil properties				
	OM (g kg^−1^)	AN (mg kg^−1^)	AP (mg kg^−1^)	AK (mg kg^−1^)	pH
T1	12.62^ab^	72.13^a^	14.23^a^	166.20^a^	5.84^a^
T2	12.20^ab^	70.68^a^	13.40^a^	165.89^a^	5.81^a^
T3	12.76^a^	69.00^a^	14.70^a^	166.85^a^	5.78^a^
R1	11.24^b^	65.34^a^	14.83^a^	152.52^a^	5.82^a^
R2	12.00^ab^	67.62^a^	13.43^a^	154.49^a^	5.80^a^
R3	12.08^ab^	68.65^a^	15.17^a^	155.96^a^	5.78^a^

OM: organic matter, AN: alkali-hydrolysable N, AP: available P, and AK: available K.

Values in each column followed by the different letters are significantly different (*P* < 0.05) according to LSD test.
